# Bone morphogenetic protein 2 is a depot-specific regulator of human adipogenesis

**DOI:** 10.1038/s41366-019-0421-1

**Published:** 2019-07-19

**Authors:** Nathan F. Denton, Mohamed Eghleilib, Sama Al-Sharifi, Marijana Todorčević, Matt J. Neville, Nellie Loh, Alexander Drakesmith, Fredrik Karpe, Katherine E. Pinnick

**Affiliations:** 1 Oxford Centre for Diabetes, Endocrinology and Metabolism, Radcliffe Department of Medicine, University of Oxford, Oxford, UK; 2 NIHR Oxford Biomedical Research Centre, Oxford University Hospital NHS Trust, Oxford, UK; 3 The MRC Weatherall Institute of Molecular Medicine, Radcliffe Department of Medicine, University of Oxford, Oxford, UK

**Keywords:** Physiology, Cell biology

## Abstract

**Background:**

Bone morphogenetic proteins (BMPs) regulate adipogenesis but it is not clear whether they influence regional adipose tissue (AT) development in humans.

**Objective:**

To characterise *BMP2* expression, BMP2-SMAD1/5/8 signalling, and BMP2′s potential effect on proliferation and adipogenesis in human subcutaneous abdominal and gluteal AT and its constituent preadipocytes.

**Methods:**

*BMP2* expression was measured in whole AT and immortalised preadipocytes via qPCR and Western blot; secreted/circulating BMP2 was measured by ELISA. The effect of BMP2 on preadipocyte proliferation was evaluated using a fluorescent assay. BMP2′s effect on adipogenesis in immortalised preadipocytes was determined via qPCR of adipogenic markers and cellular triacylglycerol (TAG) accumulation. BMP2-SMAD1/5/8 signalling was assessed in immortalised preadipocytes via Western blot and qPCR of *ID1* expression.

**Results:**

BMP2 was expressed and released by abdominal and gluteal AT and preadipocytes. Exogenous BMP2 dose dependently promoted adipogenesis in abdominal preadipocytes only; 50 ng/ml BMP2 increased *PPARG2* expression (10-fold compared to vehicle, *p* < 0.001) and TAG accumulation (3-fold compared to vehicle; *p* < 0.001). BMP2 stimulated SMAD1/5/8 phosphorylation and *ID1* expression in abdominal and gluteal preadipocytes but this was blocked by 500 nM K02288, a type 1 BMP receptor inhibitor (*p* < 0.001). Co-administration of 500 nM K02288 also inhibited the pro-adipogenic effect of 50 ng/ml BMP2 in abdominal cells; >90% inhibition of TAG accumulation (*p* < 0.001) and ~50% inhibition of *PPARG2* expression (*p* < 0.001). The endogenous iron regulator erythroferrone reduced BMP2-SMAD1/5/8 signalling by ~30% specifically in subcutaneous abdominal preadipocytes (*p* < 0.01), suggesting it plays a role in restricting the expansion of the body’s largest AT depot during energy deficiency. Additionally, a waist-hip ratio-increasing common polymorphism near *BMP2* is an eQTL associated with ~15% lower *BMP2* expression in abdominal and gluteal AT (*p* < 0.05) as well as altered adipocyte size in male abdominal AT (*p* < 0.05).

**Conclusions:**

These data implicate BMP2-SMAD1/5/8 signalling in depot-specific preadipocyte development and abdominal AT expansion in humans.

## Introduction

Several bone morphogenetic proteins (BMPs) have been identified as regulators of adipogenesis; BMP4 is a pro-adipogenic factor in human preadipocytes [1, 2] whereas BMP7 [3] promotes brown/beige adipogenesis in humans. BMP2 was originally identified as an inducer of bone and cartilage formation [4] but several reports have since implicated BMP2 in the regulation of adipogenesis in murine cell-lines; BMP2 enhanced adipogenesis in 3T3-L1 cells when co-administered with rosiglitazone [5]. Additionally, BMP2 pre-treatment promoted commitment of murine C3H10T1/2 pluripotent stem cells to the adipose lineage [6], an effect involving Smad1/5/8 and p38 kinase-mediated activation of *Pparg* [7] and *C/ebpα* [8] expression.

Much attention has focused on the role of BMP4 in human AT. Specifically, mature adipocytes secrete BMP4, which acts in a paracrine manner to enhance preadipocyte recruitment and differentiation to promote metabolically beneficial hyperplastic AT expansion [1, 9]. The apparent importance of BMP4 in the recruitment and differentiation of preadipocytes may also be true for BMP2 as these proteins share ~83% sequence homology [10]. In the context of whole-body energy metabolism, erythroferrone (ERFE) is an erythroid protein implicated in iron homeostasis, which negatively regulates hepcidin to enhance iron availability and absorption [11] by inhibiting hepatic BMP4-SMAD1/5/8 signalling [12]. Due to the structural similarity between BMP2 and BMP4, interactions with circulating factors such as ERFE could modify BMP2 action in human AT.

With body fat distribution representing a major determinant of metabolic disease risk [13], it is notable that BMP2 exerts depot-specific pro-adipogenic effects in murine cells; visceral preadipocytes are reportedly more sensitive to the pro-adipogenic effects of BMP2 compared to their subcutaneous counterparts [14]. Regional differences in BMP2-mediated Smad1/5/8 signalling may underpin this phenomenon, potentially via differential expression of endogenous inhibitors such as *Grem1* or *Smad6* [15] or depot-specific activation of transcriptional targets which regulate adipogenesis like *Id1* [16].

Emerging genetic data also suggest that developmental regulators play an important role in the determination of body fat distribution. A recent genome-wide association study (GWAS) meta-analysis notably identified a genetic locus annotated to *BMP2* (rs979012) that is significantly associated with body fat distribution (i.e. waist-hip ratio adjusted for BMI; WHRadjBMI) in men and women [17]. A significant positive association between rs979012 and BMI has also been observed [18], further suggesting that BMP2 influences AT biology. Based on the cellular data implicating BMPs in the regulation of adipogenesis and recent genetic data, we hypothesised that BMP2 exerts depot-specific effects on adipocyte development. To this end, we aimed to characterise the potential role of BMP2-SMAD1/5/8 signalling in human subcutaneous abdominal and gluteal AT.

## Material and methods

### Adipose tissue sample collection

Paired AT samples were taken from men and women in the Oxford Biobank [19] (OBB; http://www.oxfordbiobank.org.uk/). Samples were collected under local anaesthetic (1% lignocaine) from the periumbilical (subcutaneous abdominal AT) and upper buttock (gluteo-femoral AT) areas either by needle aspiration using a 12 gauge needle (yielding 250–500 mg) or using a Pro-Mag Ultra Automatic Biopsy Instrument fitted with a 14 gauge biopsy needle (yielding a ~40 mg core biopsy for histology). Samples collected by needle aspiration were either snap-frozen and stored at −80 °C for gene/protein expression analysis or used for primary preadipocyte isolation. AT core biopsies were fixed in 10% formalin for histology. DNA was extracted from whole blood by LGC Genomics (LGC Genomics, Hoddesdon, UK) and genotyping was performed using the Illumina Human Core Exome and Affymetrix UK biobank chips. Tissue donors had a median age of 45 years (range 33–53 years) and median body mass index of 25.6 kg/m^2^ (range 18.8–46.2 kg/m^2^). The taking of human AT samples was approved by the Oxfordshire Clinical Research Ethics Committee and all participants gave written informed consent.

### AT histology

AT core biopsies were embedded in paraffin wax, cut into 5 µm sections and stained with haematoxylin and eosin. Sections were viewed and photographed at ×200 magnification and adipocyte cross-sectional area was calculated using Adobe Photoshop 5.0.1 (Adobe Systems, San Jose, California, USA) and Image Processing Tool Kit (Reindeer Games, London, UK) as previously described [20]. Cell size analysis was performed on a dataset of 45 individuals (17 men, 28 women; mean age 45 years and mean BMI 26 kg/m^2^).

### Preadipocyte culture and differentiation

Primary and immortalised subcutaneous abdominal and gluteal preadipocytes were generated, maintained, and differentiated as previously described [21, 22]. Adipogenic medium was supplemented with recombinant human BMP2 (Sigma, Welwyn Garden City, UK) reconstituted in sterile PBS containing 0.1% bovine serum albumin (or vehicle). To investigate SMAD1/5/8 signalling, cells were plated and cultured for 48 h in growth medium, incubated in serum-free medium for 24 h, and then pre-treated for 30 min with serum-free medium containing K02288 (Cayman Chemicals, Ann Arbor, Michigan, USA) reconstituted in DMSO or vehicle alone. Cells were treated with BMP2 and/or K02288 in serum-free medium before harvesting after 30 min for Western blotting or 6 h for gene expression. Alternatively, cells were treated with serum-free medium supplemented with BMP2 and/or human recombinant erythroferrone (ERFE) reconstituted in sterile 20 mM Tris-HCl, 2 M MgCl_2_ (pH 7.0) before harvesting after 30 min for western blotting or 6 h for gene expression.

### Gene-expression analysis

Total RNA was extracted from abdominal and gluteal AT biopsies [23] and preadipocytes [21] and reverse transcribed as previously described. Complementary (c) DNA was synthesised from total RNA using the High Capacity cDNA Reverse Transcription Kit (Life Technologies, Warrington, UK). Quantitative (q) PCR was performed in triplicate on cDNA diluted 1/20 with Kapa Probe Fast Mastermix (Kapa Biosystems, Wilmington, Massachusetts, USA) in an 8 µl reaction. The following TaqMan Assays-on-Demand (Applied Biosystems) were used: *ADIPOQ* (Hs00605917_m1); *BMP2* (Hs00154192_m1); *BMPR1A* (Hs01034913_g1); *BMPR1B* (Hs01010965_m1); *BMPR2* (Hs00176148_m1); *SMAD6* (Hs00178579_m1); *GREM1* (Hs01879841_s1); *GREM2* (Hs03986140_s1); *ID1* (Hs03676575_s1); *PPARG2* (Hs01115510_m1); *18* *s* (Hs99999901_s1) and *PPIA* (Hs99999904_m1). Data were captured on an ABI Prism 7900HT. Relative transcript expression was calculated using the ΔΔCt relative quantification method [24] where ΔCt = Assay efficiency^(min Ct – Sample Ct)^. The ΔCt values of target genes were normalised to ΔCt values of a stably expressed reference transcript; *18* *s* was used for 14 day BMP2 treatments, *PPIA* was used for all other experiments.

### Analysis of preadipocyte proliferation

Preadipocyte proliferation was measured using the CyQuant direct cell proliferation assay (Invitrogen, Paisley, UK). Cells were seeded in growth medium on 96-well plates (8 × 10^3^ cells/well) and grown overnight. Cells were cultured in growth medium supplemented with BMP2/vehicle for 48 h, then incubated with the nucleic acid stain and background suppressor for 1 h at 37 °C. The fluorescence of each well was measured on a fluorimeter (excitation 485 nm; emission 530 nm) and the fold change in live cell number relative to control cells within the same depot was determined.

### Measurement of intracellular TAG content

Intracellular TAG content was measured in immortalised subcutaneous abdominal and gluteal preadipocytes on differentiation day 14 using an iLAB clinical analyser as previously described [22].

### Western Blotting

Whole AT biopsy lysates were prepared using an IKA homogeniser in ice-cold lysis buffer (8 M Urea; 1% SDS; 5% glycerol; 10 mM Tris-HCl; pH 6.8) and Complete EDTA-free protease inhibitor cocktail (Roche, Welwyn Garden City, UK). Whole-cell lysates were processed as previously described [22]. Conditioned medium samples were generated by incubating preadipocytes for 36 h in serum-free medium supplemented with 5 µg/ml heparin (Sigma), which protects BMP2 from degradation and extends its half-life [25]. Conditioned medium samples were concentrated on a centrifugal evaporator before blotting. Equal amounts of protein were loaded (50 µg/biopsy; 100 µg/cell lysate) and resolved by SDS-PAGE, transferred onto PVDF membranes (Bio-Rad, Watford, UK), and immunoblotted with the following antibodies: BMP2 (1:1000; NBP1-19751; Novus Biologics, Abingdon, UK); phosphorylated (p)-SMAD1/5/8 (1:1000; #13820; Cell Signalling Technology, London, UK); SMAD1/5/8 (1:500; sc-6031-R; Santa Cruz Biotechnology, Wembley, UK); β-actin (1:2000; sc1616; Santa Cruz); and α-tubulin (1:2000; ab15246; Abcam, Cambridge, UK) followed by an HRP-conjugated secondary antibody; goat anti-rabbit IgG (1:5000; 31460; ThermoFisher Scientific). Clarity enhanced chemiluminescence detection kit (Bio-Rad) was used for detection. Immunoblot images were captured on a Chemi-Doc XRS + (Bio-Rad) and analysed using ImageJ (National Institute of Health, USA).

### BMP2 ELISA

Depot-specific BMP2 release from AT was measured using arterio-venous plasma samples available from previous studies [26, 27] (nine women, age 21–53, BMI 21.5–39.3 kg/m^2^) in the fasted state) obtained as previously described [27, 28]. Briefly, blood samples were obtained from the superficial epigastric vein (draining abdominal subcutaneous AT), the saphenous vein (draining gluteal AT) and the femoral artery, following an overnight fast. All plasma samples were collected in heparin plasma tubes to minimise BMP2 degradation [25] and BMP2 concentration was analysed in duplicate by ELISA (R&D Systems).

### Data analysis and statistics

Data are presented as mean ± SEM. Statistical analyses were performed in SPSS Statistics version 22 (IBM, Sale, UK). Statistical tests used are outlined in the results section. Differences were considered statistically significant at *P* < 0.05.

## Results

### BMP2 is a paracrine factor in WAT and regulates adipogenic differentiation in a depot-specific manner

**Fig. 1:**
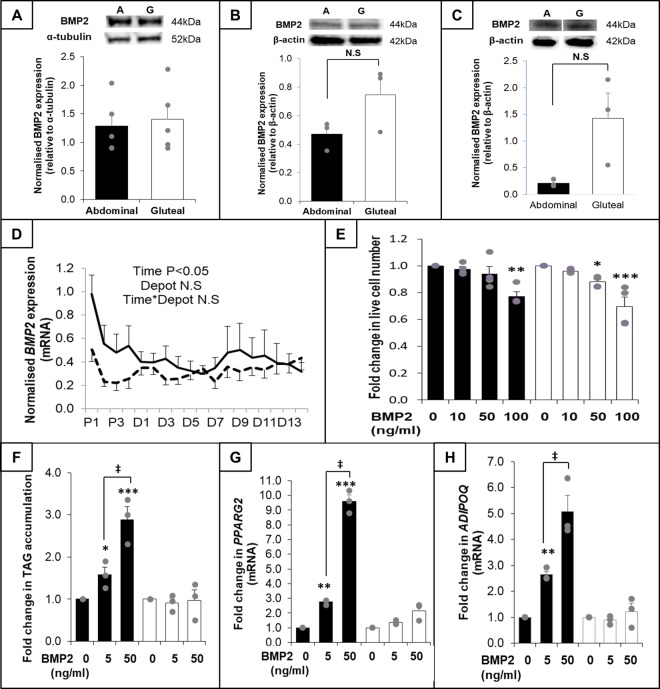
BMP2 is a paracrine factor in WAT and regulates adipogenic differentiation in a depot-specific manner. **a**, **b**, **c** Western blots and summary graphs of BMP2 and α-tubulin expression in abdominal (A; black) and gluteal (G; white) AT (A; *n* = 5 women; 37–44 years, BMI 24.1–27.1 kg/m^2^), and BMP2 and β-actin expression in proliferating (**b**) and differentiated (**c**) immortalised preadipocytes (*n* = 3). Relative protein expression data presented as mean ± SEM with individual data points overlaid (grey dots); data analysed by paired *t*-test. **d** *BMP2* mRNA expression in primary abdominal (solid line) and gluteal (dashed line) preadipocytes during 3 days of proliferation (P) followed by 14 days of differentiation (**d**) (*n* = 8; 4 men, 4 women; age 32–44 years, BMI 20.5–26 kg/m^2^). mRNA expression normalised to *PPIA*. Data analysed by repeated measures ANOVA. **e**, **f**, **g**, **h** Proliferation analysis (**e**) in immortalised preadipocytes after 48 h culture in growth medium supplemented with BMP2 (*n* = 4). Triacylglycerol (TAG) accumulation (**f**) and *PPARG2* (**g**) and *ADIPOQ* (**h**) mRNA expression in immortalised abdominal (black) and gluteal (white) preadipocytes after 14 day culture in BMP2-supplemented adipogenic medium (*n* = 3). mRNA expression normalised to 18 s. All data presented as mean fold change relative to vehicle ± SEM with individual data points overlaid (grey dots). Data analysed by ANOVA with Bonferroni *post-hoc* test (**e**, **f**, **g**, **h**); *p < 0.05, **p < 0.01, ***p < 0.001, compared to same depot vehicle; ^‡^*p* < 0.001, compared to same depot 5 ng/ml treatment

A 44 kDa band, corresponding to the inactive BMP2 pre-pro-protein [4], was readily detected in lysates of whole AT (Fig. 1a), as well as proliferating (Fig. 1b) and differentiated (Fig. 1c) immortalised preadipocytes; BMP2 expression did not vary by depot in these samples. In a larger mRNA expression panel (*n* = 190), *BMP2* was measured in subcutaneous abdominal and gluteal AT biopsies across a range of BMI (18.8–46.2 kg/m^2^) in participants from the Oxford Biobank [19]. *BMP2* mRNA was readily detectable but, consistent with the Western blot data, *BMP2* did not differ between depots (*p* = 0.97; abdominal ∆∆Ct 1.09 ± 0.026 vs. gluteal ∆∆Ct 1.08 ± 0.027). *BMP2* mRNA levels were significantly higher in female AT from the abdominal (*p* = 0.04; female ∆∆Ct 1.14 ± 0.039 vs. male ∆∆Ct 1.03 ± 0.035) and gluteal (*p* = 0.005; female ∆∆Ct 1.15 ± 0.039 vs. male ∆∆Ct 1.00 ± 0.036) depots.

A single 18 kDa band was detected in (concentrated) conditioned medium from proliferating abdominal and gluteal preadipocytes (Supplementary Fig. 1), which corresponded to the bioactive BMP2 monomer [29]. BMP2 was not detectable (i.e. <60 pg/ml) in ELISA measurements of heparinised arterio-venous samples taken across abdominal and gluteal subcutaneous AT (*n* = 9 women; age 21–53; BMI 21.5–39.3 kg/m^2^); this is consistent with studies that have reported low or undetectable BMP2 in circulating plasma samples [18, 30]. These data suggest that BMP2 principally acts as a paracrine factor in subcutaneous AT.

Further analysis of the AT mRNA expression panel dataset highlighted there was no significant correlation between BMI and *BMP2* expression in abdominal (*r*^*2*^ = 0.004, *p* = 0.40) or gluteal (*r*^*2*^ = 0.02, *p* > 0.05) AT; this was confirmed after analysing the dataset stratified according to standard BMI cut-offs (normal 18–25 kg/m^2^; overweight 25–30 kg/m^2^; and obese 30 + kg/m^2^; data not shown). *BMP2* expression was also measured via qPCR during a 14 day adipogenic time-course in primary preadipocytes (Fig. 1d) taken from healthy men and women (age 32–44 years, BMI 20.5–26 kg/m^2^). *BMP2* expression did not differ between depots but changed dynamically over time (repeated measures ANOVA; *P* < 0.05); the highest levels were detected during proliferation.

A series of dose-response experiments using exogenous BMP2 were performed using a concentration range based on the prevailing literature [5, 7]. BMP2 exerted a modest anti-proliferative effect (Fig. 1e) in preadipocytes from both depots (~15% inhibition at 100 ng/ml). Moreover, BMP2 dose-dependently enhanced adipogenesis specifically in abdominal preadipocytes as highlighted by increased TAG accumulation (Fig. 1f) as well as *PPARG2* (Fig. 1g) and *ADIPOQ* (Fig. 1h) mRNA levels but it had no effect in gluteal cells. Acute *BMP2* pre-treatment reportedly commits murine pluripotent mesenchymal stem cells to the adipose lineage and enhances their subsequent adipogenic differentiation [6]. This effect, however, did not translate to human cells (Supplementary Fig. 2A, B). These data identify BMP2 as a novel depot-specific regulator of human adipogenesis.

### BMP2 activates SMAD1/5/8 signalling and adipogenesis in preadipocytes via type 1 BMP receptors

**Fig. 2:**
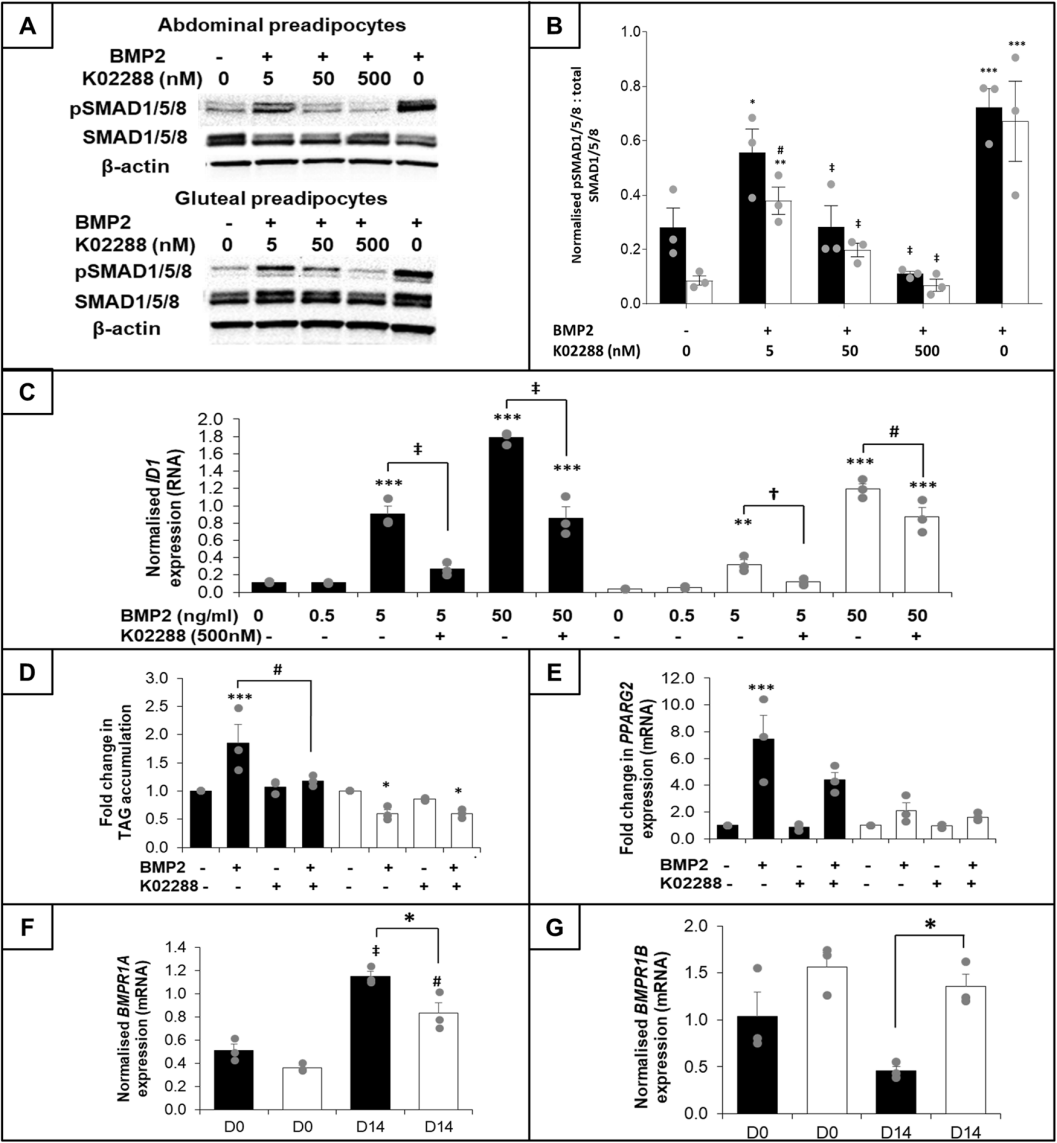
BMP2 activates SMAD1/5/8 signalling and adipogenesis in preadipocytes via type 1 BMP receptors. **a**, **b**, **c** Western blots (**a**) for phospho-(p)SMAD1/5/8, total SMAD1/5/8 and β-actin and summary graph (**b**) from immortalised abdominal (black bars) and gluteal (white bars) preadipocytes treated with 50 ng/ml BMP2 and/or K02288 for 30 min (*n* = 3; abdominal and gluteal samples run on separate gels). Data analysed by ANOVA with Bonferroni post-hoc test. *p < 0.05, **p < 0.01, ***p < 0.001, compared to same depot vehicle; ^#^*p* < 0.01, ^‡^*p* < 0.001, compared to same depot BMP2 treatment. *ID1* mRNA expression (**c**) in immortalised abdominal (black) and gluteal (white) preadipocytes after 6 h treatment with BMP2 and/or 500 nM K02288 (*n* = 3). mRNA expression normalised to *PPIA*. Data presented as mean ± SEM with individual data points overlaid (grey dots). Data analysed by ANOVA with Bonferroni *post-hoc* test. *p < 0.05, **p < 0.01, ***p < 0.001, compared to same depot vehicle; ^†^*p* < 0.05, ^#^*p* < 0.01, ^‡^*p* < 0.001, between conditions in same depot. **d**, **e** Triacylglycerol (TAG) accumulation (**d**) and *PPARG2* mRNA expression (**e**) in immortalised abdominal (black) and gluteal (white) preadipocytes after 14 day adipogenesis with 50 ng/ml BMP2 and/or 500 nM K02288 (*n* = 3). Data presented as mean fold change relative to vehicle ± SEM with individual data points overlaid (grey dots). Data analysed by ANOVA with Bonferroni *post-hoc* test; *p < 0.05, **p < 0.01, ***p < 0.001, compared to same depot vehicle; ^†^*p* < 0.05, ^#^*p* < 0.01, ^‡^*p* < 0.001, between indicated conditions in same depot. **f**, **g** *BMPR1A* (**f**) and *BMPR1B* (**g**) mRNA expression in immortalised abdominal (black) and gluteal (white) preadipocytes on day (**d**) 0 and 14 of adipogenesis (*n* = 3). Data presented as mean normalised expression ± SEM with individual data points overlaid (grey dots). mRNA expression normalised to *PPIA*. Data analysed by ANOVA with Bonferroni *post-hoc* test (**c**, **d**); *p < 0.05, between depots at the same time-point; ^#^*p* < 0.01, ^‡^*p* < 0.001, compared to D0 in same depot

We next characterised BMP2-SMAD1/5/8 signalling in immortalised abdominal and gluteal preadipocytes. Treatment with 50 ng/ml BMP2 strongly induced SMAD1/5/8 phosphorylation in proliferating abdominal and gluteal preadipocytes within 30 min of administration (Fig. 2a, b, lane 5); no depot-specific difference in basal (*p* = 0.063) or BMP2-induced SMAD1/5/8 phosphorylation (*p* = 0.607) was detected. This effect was mediated via type 1 BMP receptors as pre-treatment and co-administration with K02288—an inhibitor of type 1 BMP receptor kinase activity [31]—dose dependently inhibited BMP2-induced SMAD1/5/8 phosphorylation in abdominal and gluteal cells (Fig. 2a, b, lanes 2–4); complete inhibition was achieved with 500 nM K02288 (Fig. 2a, b, lane 4).

Treatment with BMP2 for 6 h dose-dependently induced *ID1* expression in preadipocytes from both depots (Fig. 2c). *ID1* is a transcriptional target of BMP2 regulated by SMAD1 and SMAD4 activity [32] implicated in the regulation of adipogenesis[16]; higher *ID1* expression was induced in abdominal cells with 5 ng/ml (0.9 ± 0.1 vs. 0.3 ± 0.1, *p* < 0.001) and 50 ng/ml BMP2 (1.8 ± 0.04 vs. 1.2 ± 0.06, *p* < 0.001). Activation of *ID1* expression was blocked by pre-treatment and co-administration with 500 nM K02288 in abdominal and gluteal cells. The depot-specific pro-adipogenic effect of BMP2-required type 1 BMP receptor-mediated activation of SMAD1/5/8 signalling as co-administration of 500 nM K02288 completely blocked the BMP2-induced TAG accumulation in abdominal preadipocytes (Fig. 2d) and partially reduced *PPARG2* expression (Fig. 2e). K02288 treatment alone did not affect preadipocyte differentiation in either depot.

**Fig. 3:**
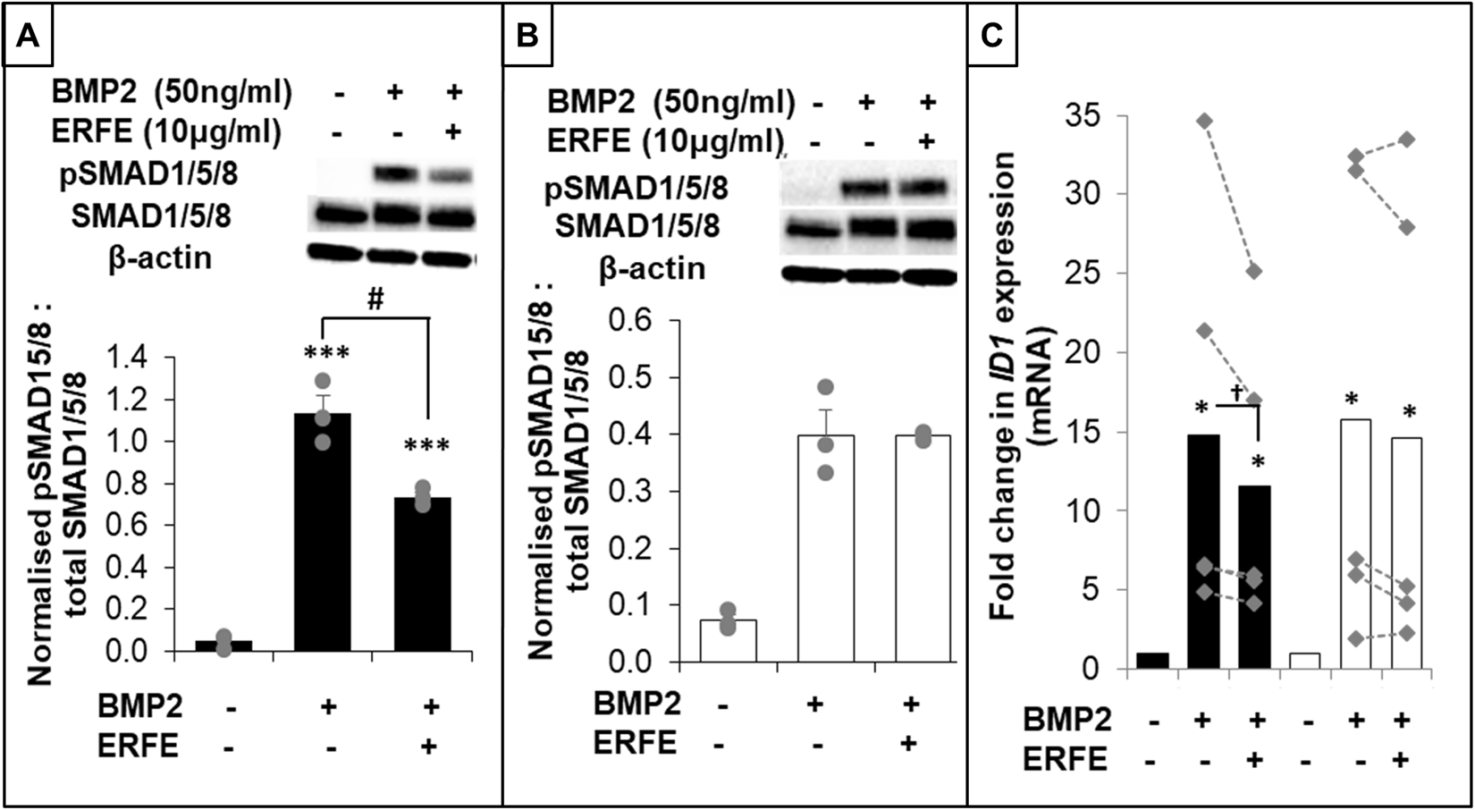
Erythroferrone (ERFE) is a depot-specific regulator of BMP2 action in preadipocytes. **a**, **b** Western blots and summary graphs for phospho-(p)SMAD1/5/8, total SMAD1/5/8 and β-actin in immortalised abdominal (**a**) and gluteal (**b)** preadipocytes after 30 min treatment with 50 ng/ml BMP2 and/or 10 µg/ml erythroferrone (ERFE) (*n* = 3; abdominal and gluteal samples run on separate gels). Data presented as mean ± SEM with individual data points overlaid (grey dots) and analysed by ANOVA with Bonferroni post-hoc test; ***p < 0.001, compared to same depot vehicle; ^#^*p* < 0.01, compared to same depot BMP2 treatment. **c** *ID1* mRNA expression in immortalised preadipocytes treated with BMP2 and/or ERFE for 6 h (*n* = 5). Data presented as mean fold change (bars) with individual paired data points overlaid (grey diamonds). Data analysed by ANOVA with post-hoc Wilcoxon Signed-Rank Test; *p < 0.05, compared to same depot vehicle; ^†^*p* < 0.05, between conditions in same depot

To identify potential mechanism(s) underlying the depot-specific effects of BMP2 on adipogenesis, the expression of SMAD1/5/8 signalling pathway components was analysed. While showing no differences at day 0, *BMPR1A* expression was significantly higher in abdominal preadipocytes on day 14 (Fig. 3f) whereas *BMPR1B* expression was significantly lower in abdominal preadipocytes on day 14 (Fig. 3g) compared to gluteal cells. Expression of other signalling components such as *BMPR2* and the endogenous inhibitor *SMAD6* [15] did not vary according to depot or time (data not shown).

### Erythroferrone (ERFE) is a depot-specific regulator of BMP2 action in preadipocytes

ERFE is an erythroid protein implicated in iron homeostasis, which negatively regulates hepcidin to enhance iron availability and absorption [11] by inhibiting hepatic BMP4-SMAD1/5/8 signalling [12]. Given the high degree of structural similarity between BMP2 and BMP4 [10], it was investigated whether ERFE modifies BMP2-SMAD1/5/8 signalling in abdominal and gluteal preadipocytes. It was found that co-administration of ERFE (10 μg/ml) inhibited BMP2-induced SMAD1/5/8 phosphorylation by ~30% in abdominal preadipocytes (Fig. 3a) but had no effect in gluteal cells (Fig. 3b). ERFE also reduced BMP2-mediated activation of *ID1* expression by ~20% in abdominal but not in gluteal preadipocytes (Fig. 3c).

### The rs979012/rs2145270 genetic locus is an eQTL for BMP2 expression in AT and is associated with adipocyte size in male subcutaneous abdominal AT

A recent GWAS meta-analysis identified a genome-wide significant association between rs979012 (125 kb upstream of *BMP2*) and WHRadjBMI [17]. This result prompted us to investigate whether this locus represented an expression quantitative trait locus (eQTL) for *BMP2* in AT; it is noted that significant eQTL signals have not been detected for this SNP in the GTex dataset. The previously described dataset in which *BMP2* mRNA levels were measured in subcutaneous abdominal and gluteal AT biopsies from men and women (*n* = 190) across a range of BMI (18.8–46.2 kg/m^2^) in participants from the Oxford Biobank [19] was used for eQTL analysis.

LDproxy (NIHR; https://analysistools.nci.nih.gov/LDlink/) was used to identify rs2145270 (C/T) as a suitable proxy (*r*^*2*^ = 0.96, D’ = 1.000) for rs979012 (T/C). As these loci also associate with height [33], potential differences in height between genotypes were examined to identify possible anthropometric confounders that could interfere with estimation of regional fat distribution, but associations with height were not detected. Using a recessive model, correcting for age, sex, and BMI, it was found that *BMP2* mRNA levels in abdominal (0.94 ± 0.07 vs. 1.11 ± 0.03; *p* = 0.042) and gluteal (0.94 ± 0.08 vs. 1.095 ± 0.03; *p* = 0.028) AT were significantly lower (~15%) in homozygous carriers of the rare C allele (*n* = 24) at rs2145270 compared to their CT/TT counterparts (*n* = 165), indicating the presence of an eQTL. It remains unclear whether the expression of BMP2-responsive genes was also affected.

The structure of AT was also examined to investigate whether the rs979012/rs2145270 locus was associated with an adipocyte size phenotype. Abdominal and gluteal adipocyte data were split by sex and grouped according to rs2145270 genotype, with individuals homozygous for the abdominal fatness-increasing C allele being compared to those homozygous for the common T allele. Cells were binned according to their area (reported as µm^2^) and the proportion of each size range to the total population counted was calculated. A small but significant shift in cell size distribution was observed in male abdominal AT (Table 1) in which the CC genotype associated with increased abdominal fatness was also associated with more large adipocytes (5000–10,000 µm^2^) at the expense of smaller (<500 µm^2^) cells. This is also reflected in the mean adipocyte size in male abdominal AT (*p* < 0.05; CC mean 3538 µm^2^ ± 233 vs. TT mean 3251 µm^2^ ± 206). No significant differences in adipocyte size were observed between genotypes in female abdominal AT (Table 1) or gluteal AT from either sex (data not shown).

**Table 1:** The rs979012/rs2145270 genetic locus is associated with adipocyte size in male subcutaneous abdominal AT

Cell Area (µm^2^)	Male genotypes		*p*	Female genotypes		*p*
	CC	TT		CC	TT	
<500 (small)	5.1 (0.9)	7.6 (0.8)	*0.031	6.8 (0.9)	6.7 (0.8)	0.77
500–5000 (medium)	69.3 (2.6)	69.6 (2.2)	0.087	66.9 (3.8)	66.0 (3.1)	0.22
5000–10000 (large)	23.1 (2.7)	20.4 (2.2)	*0.038	22.5 (2.8)	22.1 (2.3)	0.16
>10000 (very large)	2.5 (0.6)	2.4 (0.5)	0.2	3.8 (1.5)	5.2 (1.3)	0.38
*n*	7	10		10	18	

Male and female subcutaneous abdominal adipocyte size analysis according to rs2145270 genotype. Data expressed as mean proportion (%) of total cells counted and reported as estimated marginal means (SEM). Data analysed using ANCOVA (corrected for age and BMI). *p < 0.05.

Size analysis of primary male and female subcutaneous abdominal adipocytes (*n* = 28 women, 17 men; age 30–66 years; BMI 20.6–41.5 kg/m^2^) according to rs2145270 genotype. Data expressed as mean proportion (%) of total cells counted and reported as estimated marginal means (SEM). Data analysed using ANCOVA (corrected for age and BMI). *p < 0.05.

## Discussion

Identifying depot-specific factors with regulatory roles in the recruitment, commitment and differentiation of WAT preadipocytes is fundamental for understanding regional AT expansion. BMP2, a member of the TGF-β growth factor superfamily, has classically been studied in the context of bone/cartilage biology [34]. Here we present compelling evidence that BMP2 is also a modulator of preadipocyte biology, which exerts striking depot-specific effects on the development of abdominal and gluteal adipocytes.

The results from this study demonstrate for the first time that bioactive BMP2 is expressed, processed, and secreted by human abdominal and gluteal preadipocytes, suggesting BMP2 acts as a paracrine factor within subcutaneous AT. BMP2 was undetectable in arterio-venous samples collected across subcutaneous abdominal and femoral AT from females in the fasted state. It is currently unclear if there are sex differences in BMP2 release or if it is affected by feeding. Other studies have reported similarly low levels of circulating BMP2 [18, 30] so it would seem that adipose-derived BMP2 has a limited endocrine role. Elevated BMP2 serum concentrations have been reported in patients with type 2 diabetes and moderate obesity [18]. However, the regulation of *BMP2* expression in mesenchymal cells is highly complex [35] and it remains unclear what contribution subcutaneous AT makes to systemic BMP2 concentrations, or whether BMP2 is involved in tissue cross-talk in health and/or disease.

Several transcriptomic studies have shown that many genes which regulate developmental processes, particularly gene transcription, are differentially expressed between abdominal and gluteal AT [36, 37] but this does not seem to be the case for BMP2. No depot-specific expression pattern (mRNA or protein) was detected in whole AT or proliferating/differentiating preadipocytes from these depots, although it has been reported that *BMP2* mRNA levels are higher in visceral compared to subcutaneous abdominal AT [18]. We found that *BMP2* expression did not vary in male or female subcutaneous abdominal or gluteal AT in a BMI-dependent manner; the former result contrasts with a previous report that *BMP2* expression is elevated in obese subcutaneous (abdominal) AT compared to AT from normal weight individuals [18]. Such a difference may reflect differences in the datasets, with the other study utilising a larger set of AT samples that represented a wider range of BMI that included more morbidly obese individuals with/without type 2 diabetes. Additional studies are also needed to elucidate whether BMP2 protein expression varies between AT depots in relation to genotype, BMI, WHR, and sex. While the synthesis of BMP2 did not differ between abdominal and gluteal AT, this did not preclude these depots from exhibiting depot-specific responses to BMP2 upon exposure.

In our in vitro studies we observed that persistent BMP2 treatment produced a striking depot-specific effect that strongly increased adipogenic gene expression and TAG accumulation in abdominal preadipocytes but had a neutral or modest inhibitory effect on gluteal cells. The pro-adipogenic action of BMP2 aligns with previous data from murine studies in which BMP2 promoted adipogenesis via increased *PPARG* expression and transcriptional activity driven by Smad1/5/8 and p38 kinase signalling [7]. Murine data suggest that BMP2-Smad1 signalling positively regulates *Pparg* expression via the transcription factor *Ahnak* [38]. Consistent with our findings, *AHNAK* is more highly expressed in abdominal compared to gluteal AT in humans [37]. Our data identify BMP2 as a novel endogenous regulator of adipogenesis that acts to increase PPARG in a depot-specific manner, meaning BMP2 has the potential to direct regional AT expansion and influence metabolic health in humans [13].

Despite the striking depot-specific effect on adipogenesis, BMP2 activated SMAD1/5/8 via type 1 BMP receptors to a similar extent in abdominal and gluteal preadipocytes. However, the signal was not transduced equally in both depots as *ID1* expression, a downstream SMAD1/5/8 target gene implicated in the regulation of adipogenesis [16], was induced to a lesser extent in gluteal cells. This effect may derive from differential expression of BMP2-SMAD1/5/8 signalling components such as type 1 BMP receptor subtypes.

Previous data indicate that BMPR1A is pro-adipogenic whereas BMPR1B is pro-osteogenic [39]; human adipose-derived stem cells (hASCs) enriched for *BMPR1A* expression exhibit a greatly enhanced adipogenic capacity compared to their *BMPR1A*-null counterparts [40]. Conversely, hASCs enriched for *BMPR1B* expression display an enhanced osteogenic capacity in vitro and in vivo [41]. In this study we demonstrated that in vitro differentiated abdominal preadipocytes expressed higher levels of *BMPR1A* and lower levels of *BMPR1B* compared to gluteal cells. This regional variation in BMP receptor subtype expression is consistent with the depot-specific pro-adipogenic effect of BMP2 being restricted to abdominal cells. However, it was beyond the scope of the current study to determine whether differential expression of BMP receptor subtypes underpins BMP2′s depot-specific actions.

As the modest anti-adipogenic effect of BMP2 in gluteal cells was not blocked by the type 1 BMP receptor kinase inhibitor K02288, it is possible that non-SMAD1/5/8 signalling activity contributes to regional variation in BMP2 action. Other potential mediators of depot-specific BMP2 action include extracellular BMP antagonists like GREM1 and GREM2, which bind BMPs to inhibit ligand-receptor interactions and downstream signalling [42]. GREM1 is a negative regulator of BMP4-mediated adipogenesis [9] and is more highly expressed in gluteal AT [37]. Our data also highlighted that ERFE selectively inhibited BMP2-SMAD1/5/8 signalling in abdominal preadipocytes. These data suggest that energy deficiency signals from energy-sensing organs could modify BMP2 action to ameliorate the expansion of the largest fat-storing adipose organ in the human body (i.e. subcutaneous abdominal AT) to potentially influence body fat distribution and overall metabolic health [13].

There is also mounting appreciation that epigenetic mechanisms play a crucial role in the regulation of gene expression and may therefore contribute to the biological responses of cells to BMP2 and potentially other growth factors. Recent data indicate that BMP4 regulates the expression and activation of *ZNF423*—the human homologue of the transcription factor *Znf423*, which co-ordinates SMAD-dependent activation of *Pparg* expression [43]—in subcutaneous abdominal preadipocytes by modifying DNA methylation [44]. As abdominal and gluteal preadipocytes exhibit distinct epigenetic profiles [37], and cell-specific patterns of chromatin accessibility affecting SMAD binding have been detected [45], regional variation in epigenetic profiles may underpin the depot-specific adipogenic response of preadipocytes upon BMP2 treatment. Epigenetic mechanisms may also explain how depot-specific biological responses to growth factors such as BMP2 are transferred between different cell generations.

We also identified that the rs979012/rs2145270 GWAS locus associated with body fat distribution [17] represents an eQTL for *BMP2* expression in subcutaneous abdominal and gluteal AT, although it is unclear whether the expression of BMP2-responsive genes was also affected. Additionally, males homozygous for the WHRadjBMI-increasing C allele displayed a modestly shifted cell size distribution, which favoured larger cells at the expense of smaller cells in male abdominal AT. It is speculated that the presence of larger adipocytes in abdominal AT reflects fewer new preadipocytes being recruited for differentiation by the genetically-determined lower BMP2 signalling in the organ. As larger adipocytes, particularly in the upper body, are usually indicative of adipose dysfunction and insulin resistance [46, 47], this morphological shift may mean that homozygous male, but not female, carriers of the C allele at the rs2145270 locus are at increased risk of developing metabolic disease, although this requires further examination.

Overall, these data identify BMP2 as a novel, depot-specific regulator of adipogenesis in human subcutaneous AT. They also strongly implicate BMP2-SMAD1/5/8 signalling in the determination of body fat distribution in humans and emphasise the importance of comparative studies examining multiple AT depots.

## Supplementary information

Supplementary Figure 1

Supplementary Figure 2
