# Correction to: Bone morphogenetic protein 2 is a depot-specific regulator of human adipogenesis

**DOI:** 10.1038/s41366-019-0475-0

**Published:** 2019-10-22

**Authors:** Nathan F. Denton, Mohamed Eghleilib, Sama Al-Sharifi, Marijana Todorčević, Matt J. Neville, Nellie Loh, Alexander Drakesmith, Fredrik Karpe, Katherine E. Pinnick

**Affiliations:** 1 Oxford Centre for Diabetes, Endocrinology and Metabolism, Radcliffe Department of Medicine, University of Oxford, Oxford, UK; 2 NIHR Oxford Biomedical Research Centre, Oxford University Hospital NHS Trust, Oxford, UK; 3 The MRC Weatherall Institute of Molecular Medicine, Radcliffe Department of Medicine, University of Oxford, Oxford, UK

**Keywords:** Physiology, Cell biology

**Correction to: International Journal of Obesity**

10.1038/s41366-019-0421-1; Published online 19 July 2019

We erroneously published the original Article with an incorrect Copyright line. This has been updated in the XML, PDF and HTML versions of this Article.

