# Supplementary figures and images for: Bone morphogenetic protein 2 is a depot-specific regulator of human adipogenesis
Source: Int J Obes (Lond). 2019 Jul 19;43(12):2458–68. doi: 10.1038/s41366-019-0421-1 (PMC6892741; doi:10.1038/s41366-019-0421-1)

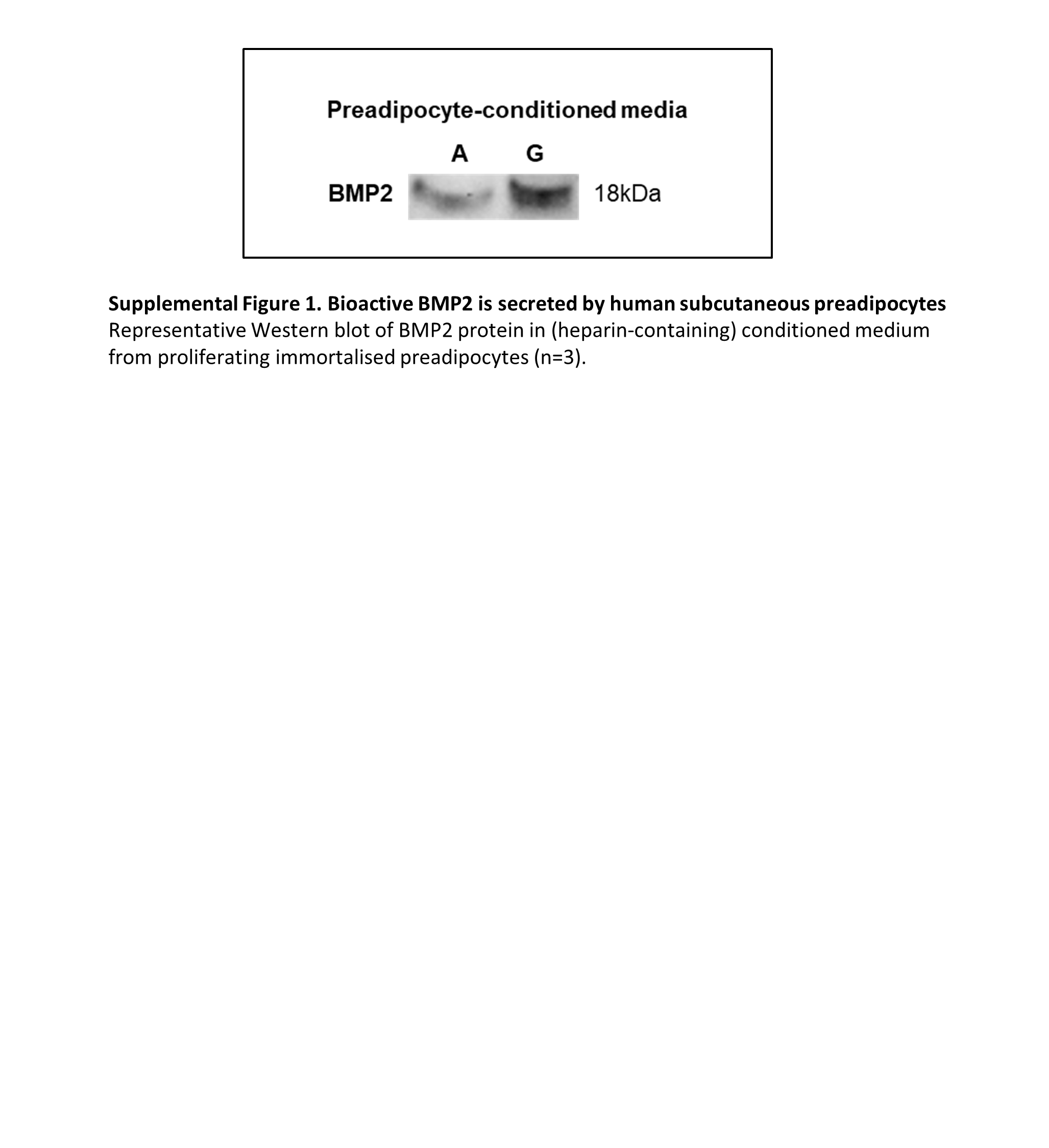

Supplement: Supplementary file 1 — Supplementary Figure 1 [file 41366_2019_421_MOESM1_ESM.tif]

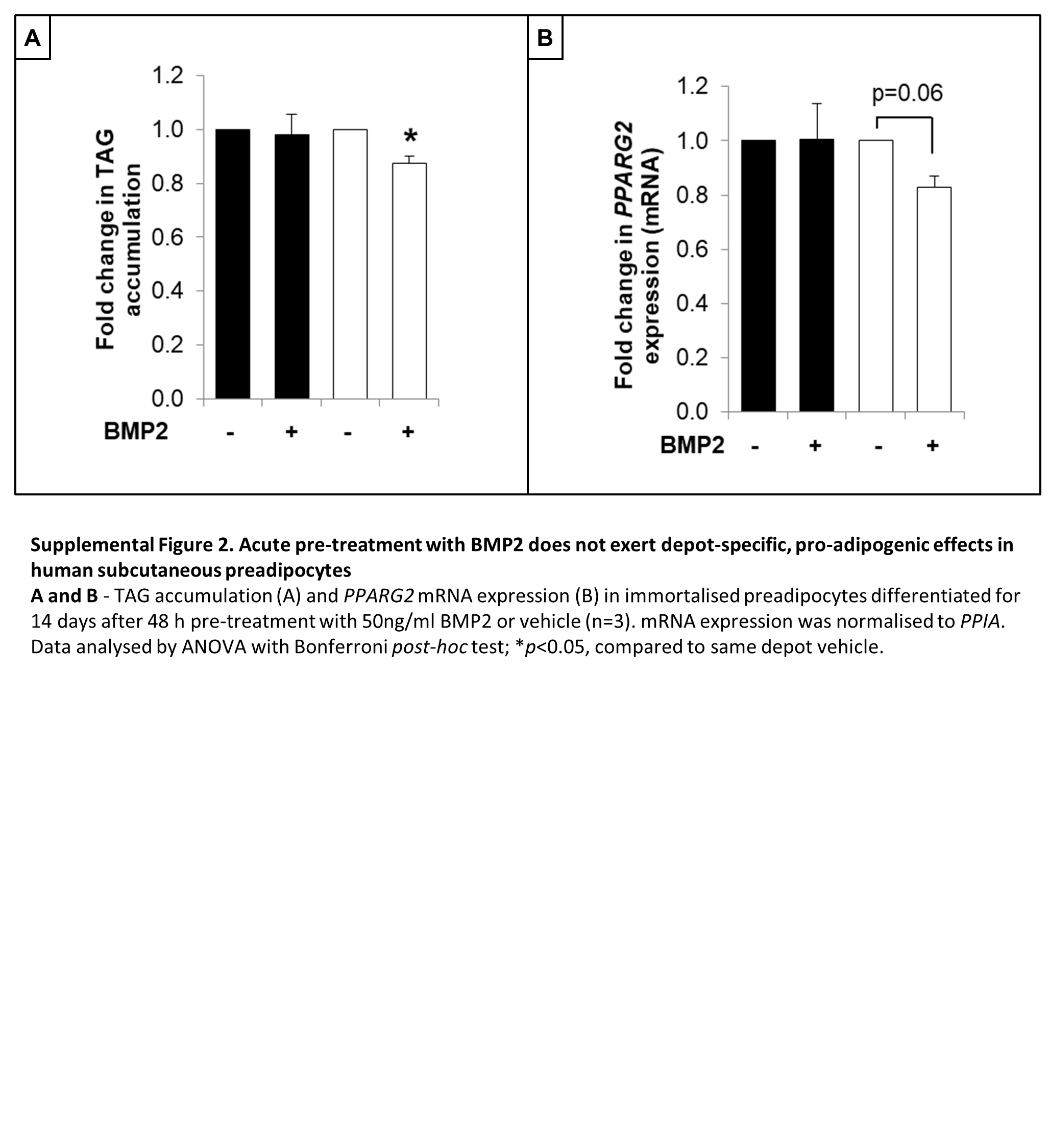

Supplement: Supplementary file 2 — Supplementary Figure 2 [file 41366_2019_421_MOESM2_ESM.tif]
